# Prevalence of Neuropathic Component in Post-COVID Pain Symptoms in Previously Hospitalized COVID-19 Survivors

**DOI:** 10.1155/2022/3532917

**Published:** 2022-03-16

**Authors:** Manuel Herrero-Montes, César Fernández-de-las-Peñas, Diego Ferrer-Pargada, Sandra Tello-Mena, Ignacio Cancela-Cilleruelo, Jorge Rodríguez-Jiménez, Domingo Palacios-Ceña, Paula Parás-Bravo

**Affiliations:** ^1^Departamento de Enfermería, Universidad de Cantabria, Santander 39008, Spain; ^2^Instituto de Investigación Sanitaria Valdecilla (IDIVAL), Grupo de Investigación en Enfermería, Santander 39008, Spain; ^3^Department of Physical Therapy, Occupational Therapy, Physical Medicine and Rehabilitation, Universidad Rey Juan Carlos (URJC), Madrid 28922, Spain; ^4^Servicio de Neumología, Hospital Universitario Marqués de Valdecilla, Santander 39008, Spain

## Abstract

**Objectives:**

To investigate the prevalence of neuropathic pain symptoms and to analyze the correlation between neuropathic symptoms with pain-related, psychological, and cognitive variables in COVID-19 survivors exhibiting “de novo” post-COVID pain.

**Methods:**

Seventy-seven (*n* = 77) previously hospitalized COVID-19 survivors presenting with post-COVID pain completed demographic (such as age, height, and weight), pain-related (the duration and intensity of pain), psychological (depressive/anxiety levels), and cognitive (catastrophizing and kinesiophobia) variables. The Self-Report Leeds Assessment of Neuropathic Symptoms and Signs (S-LANSS) questionnaire was also assessed. After conducting multivariable correlation analyses, a stepwise multiple linear regression model was performed to identify S-LANSS predictors.

**Results:**

Participants were assessed a mean of 6.0 (SD 0.8) months after hospital discharge. Nineteen (24.6%) exhibited neuropathic pain symptoms (S-LANSS score≥12 points). The S-LANSS score was positively associated with the duration of post-COVID pain (*r*: 0.262), anxiety levels (*r*: 0.275), and kinesiophobia level (*r*: 0.291) (all, *P* < 0.05). The stepwise regression analysis revealed that 12.8% of the S-LANSS variance was just explained by kinesiophobia.

**Conclusion:**

This study found that almost 25% of previously hospitalized COVID-19 survivors with “de novo” post-COVID pain reported a neuropathic pain component. The presence of neuropathic pain symptomatology was associated with more anxiety and kinesiophobia, but only kinesiophobia level was significantly associated explaining 12.8% of the variance of the S-LANSS score.

## 1. Introduction

Musculoskeletal pain (myalgia) is one of the most common symptoms experienced during the acute phase of severe acute respiratory syndrome coronavirus-2 (SARS-CoV-2) infection [1, 2]. In addition, pain is also present in up to 18% of individuals experiencing post-COVID symptoms during the first year after infection [3]. Characterization of post-COVID pain could help for better understand potential mechanisms and orientate personalized-tailored treatments. Although post-COVID pain resembles musculoskeletal features [4], neuropathic pain has been also described as a post-COVID sequelae [5]. It is possible that post-COVID pain exhibits both musculoskeletal and neuropathic pain features. In fact, discrimination between nociceptive, neuropathic, and nociplastic pain represents a current challenge for clinicians [6].

Preliminary evidence suggests the presence of neuropathic pain in individuals exhibiting post-COVID pain. Vaz et al. [7] described the development of complex regional pain syndrome (a neuropathic pain condition) in a patient who had survived COVID-19. Similarly, McWilliam et al. [8] reported neuropathic pain as a post-COVID sequelae. A cohort study recently reported that almost 25% of patients with post-COVID pain exhibit neuropathic symptoms; however, this study collected self-reported symptoms throughout a telephonic interview [9].

Screening self-reported questionnaires help clinicians identify neuropathic pain symptoms, particularly in those with complex pain conditions [10]. The Self-Report Leeds Assessment of Neuropathic Symptoms (S-LANSS) is a self-rated questionnaire commonly used for assessing the presence of neuropathic pain [10]. In fact, it has the proper sensitivity and specificity scores to be compared with nociceptive pain [11]. No study has used the S-LANSS for determining the presence of neuropathic symptoms in COVID-19 survivors. Accordingly, the aims of this study were as follows: 1) to investigate the prevalence of neuropathic pain symptoms using the S-LANSS, and 2) to analyze the correlation between S-LANSS and pain-related, psychological, and cognitive variables in previously hospitalized COVID-19 survivors exhibiting “de novo” post-COVID pain.

## 2. Methods

### 2.1. Study Design

An observational cross-sectional cohort study following the Strengthening the Reporting of Observational Studies in Epidemiology (STROBE) guidelines [12] was conducted (Suppl. File). This study was approved by the Institutional Ethics Committee of IDIVAL Cantabria (code 2020.416). Participants were informed of the study, and all provided their informed consent prior to their inclusion.

### 2.2. Participants

Individuals who had recovered from SARS-CoV-2 infection were diagnosed with a real-time reverse transcription-polymerase chain reaction (PCR) assay of nasopharyngeal/oral swab samples and the presence of radiological findings and hospitalized at an urban hospital in Santander (Spain) were recruited. They were included if they presented with the following: (1) “de novo” pain symptoms compatible with a diagnosis of chronic primary musculoskeletal pain as defined by the International Association for the Study of Pain [13]; (2) pain symptoms should have started after COVID-19 and be present for at least three consecutive months after hospital discharge; and (3) absence of medical conditions which could best explain the presence of pain, such as arthritis. They were excluded if they reported a preexisting history of pain or any preexisting medical comorbidity explaining the presence of pain symptomatology.

### 2.3. Collection Data

Patients attending a specific post-COVID unit at an urban hospital (Hospital Universitario Marqués de Valdecilla) in Santander (Spain) from 1st June 2021 to 31^st^ October 2021 were recruited. Individuals reporting pain as their main post-COVID symptom were invited to participate if they fulfilled the inclusion criteria. Demographic (including age, weight, and height) and clinical (pain intensity, numerical pain rating scale (NPRS): 0–10, and location of pain) data were collected. Additionally, the following self-reported questionnaires were also collected: S-LANSS, the Hospital Anxiety and Depression Scale (HADS), the Pain Catastrophizing Scale (PCS), and the 11-item short-form of the Tampa Scale for Kinesiophobia (TSK-11).

### 2.4. Neuropathic Pain Assessment–The S-LANSS

In this study, the Spanish version of the S-LANSS was used [14]. The S-LANSS questionnaire uses a binary response where subjects confirm whether they experience different symptoms to classify them into a predominantly or nonpredominantly neuropathic origin [14]. The Spanish version of the S-LANSS has shown good sensitivity, internal consistency, and validity [14]. Its score ranges from 0 to 24, where subjects obtaining ≥12 points are susceptible to neuropathic pain [14].

### 2.5. Anxiety and Depressive Levels

The Spanish version of the HADS was used to determine anxiety and depressive levels [15]. We included both scales, the first assessing anxiety symptoms (HADS-A, 7-items, 0–21 points) and the second assessing depressive symptoms (HADS-D, 7-items, 0–21 points). Higher scores suggest more anxiety/depressive levels [15]. We considered those cut-off scores recommended for the Spanish population indicative of anxiety (HADS-A ≥ 12 points) or depressive (HADS-D ≥10 points) symptoms [16].

### 2.6. Cognitive Variables

The Spanish version of the PCS was used to assess pain catastrophizing [17]. It includes 13-items evaluating rumination (constant worry and inability to inhibit thoughts about pain), magnification (exaggeration of unpleasantness of painful situations and expectations of negative consequences), and despair (inability to face pain) aspects. Items are answered in a 5-point Likert scale where 0 means “never” and 4 means “always” (total score 0–52 points) [17].

The Spanish version of the TSK-11 was used to determine the fear of movement perceived by the patient [18]. This questionnaire includes 11 items where the patient chooses in a 4-point Likert scale how much he/she agrees/disagrees with each item, being 1 “complete disagreement” and 4 “complete agreement” (total score from 0 to 44 points) [18].

### 2.7. Statistical Analysis

Descriptive analyses (means and standard deviations (SD)) were used to describe the sample. The Kolmogorov–Smirnov test revealed that quantitative data exhibited a normal distribution. Between-group differences based on the S-LANSS score (<12 or ≥12 points) were assessed with the independent Student's *t*-tests. Correlations between the S-LANSS (dependent variable) score with the remaining variables were assessed by using Pearson's correlation coefficients (r). The correlation coefficients were also used to identify multicollinearity and shared variance between the variables (when *r* > 0.8). All statistically significant variables associated with the S-LANSS were included in a stepwise multiple hierarchical linear regression model to assess those independent variables contributing significantly to its variance, except variables showing multicollinearity. The significance criterion of the critical F value for entry into the regression equation was set at *P* < 0.05. Changes in adjusted *R*^2^ were reported after each step of the regression model.

## 3. Results

**Figure 1:**
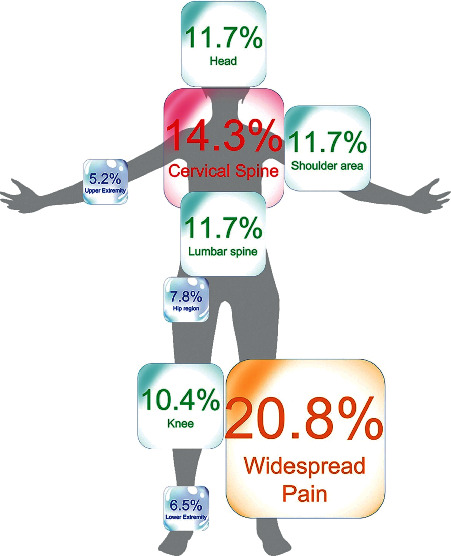
Location of post-COVID pain in previously hospitalized COVID-19 survivors (*n* = 77).

One hundred and fifty (*n* = 150) individuals attending the post-COVID unit from 1st June 2021 to 31st October 2021 were screened for eligibility criteria. Seventy-three (48%) were excluded because their main post-COVID symptom was respiratory (*n* = 45) and not pain, or because of the presence of previous pain symptoms (*n* = 28). Finally, 77 patients (37.6% women; age: 60 and SD: 11.5 years) satisfied all inclusion criteria. Participants were assessed a mean of 6.0 ± 0.8 months after hospital discharge.  Table 1 details the characteristics of the sample.  Figure 1 graphs the location of post-COVID pain symptoms in the sample.

The mean score on the S-LANSS was 7.7 (SD: 6.5), where 19 (24.6%) individuals had a score of ≥12/24 and 58 (75.3%) had a score of <12/24. Subjects with neuropathic pain symptoms (S-LANSS≥12 points) showed higher catastrophizing or kinesiophobia levels (*P* < 0.05) than those without neuropathic pain symptoms (S-LANSS < 12 points) (Table 1).

The bivariate correlation analyses are reported in Table 2. The S-LANSS score was positively associated with time with pain symptoms, anxiety levels, and higher kinesiophobia levels (all, *P* < 0.05). Significant positive associations were also found among psychological and cognitive variables (*r* values ranging from .321 to .639).

The stepwise regression analysis revealed that only kinesiophobia was a significant predictor of S-LANSS explaining 12.8% of the variance of the total score (r2 adjusted: 0.128, *B* = 0.358, 95% CI: 0.09–2.736, *t* = 2.736, and *P* = 0.009).

## 4. Discussion

To the best of the authors' knowledge, this is the first cohort study investigating the prevalence of neuropathic pain symptomatology using a validated self-reported questionnaire such as the S-LANSS, in COVID-19 survivors with “de novo” post-COVID pain. We found that almost 25% of previously hospitalized COVID-19 survivors exhibiting post-COVID pain reported a neuropathic component. The presence of neuropathic pain symptoms was also associated with higher levels of anxiety and kinesiophobia, but only kinesiophobia was able to explain a small part of the variance of the S-LANSS score (12.8%).

The prevalence of neuropathic pain in our sample of COVID-19 survivors (25%) was similar to the rate provided by Oguz-Akarsu et al. [9] by using a telephone interview but self-reported. Current prevalence data of neuropathic symptoms in COVID-19 survivors (25%) is higher than the prevalence of neuropathic symptoms reported by a nationwide study (6.9%) in people with chronic pain [19], supporting the expected increase in the prevalence of neuropathic pain due to COVID-19 [5]. The neuroinvasive potential of the SARS-CoV-2 virus explaining the presence of neuropathic pain symptoms in COVID-19 survivors can be explained by the high expression of angiotensin converting enzyme-2 (ACE2) receptors detected within nervous system cells including neurons and microglia in the spinal dorsal horn [20]. Additionally, the SARS-CoV-2 cytokine and interleukin associated storms may lead to sensitization of peripheral and central pain pathways [21, 22], promoting the development of chronic pain. In such a scenario, the SARS-CoV-2 virus may trigger different mechanisms that, in predisposing individuals, would lead to the development of neuropathic pain. Nevertheless, the role of ACE2 receptors on peripheral small-fiber sensory neurons is still not known [23].

We also identified that anxiety and kinesiophobia levels were associated with neuropathic pain, although just kinesiophobia was significant in the logistic regression. Hruschak et al. [24] found that chronic pain patients with higher catastrophizing levels were at a higher risk of social isolation, and accordingly more pain, during the main COVID-19 outbreak. It would be expected that patients with neuropathic pain would exhibit more kinesiophobia; nevertheless, the logistic regression identified that the contribution of kinesiophobia to the S-LANSS score was small, in agreement with previous studies [25].

Precision medicine implies that patient education, management, and treatment must be adapted to the pain phenotype of each patient. For instance, implementation of telemedicine for the management of those identified factors associated with neuropathic pain, such as anxiety or kinesiophobia, could be effectively applied for the management of post-COVID pain [26]. As neuropathic pain is related to cognitive/emotional factors including catastrophizing, depressive or anxiety levels, kinesiophobia, stress, or maladaptive illness perception, clinicians should consider individually tailored multimodal treatments combining pain neuroscience education with physical therapy and stress management as well as medication intake in this population.

Finally, the current study has some limitations. First, current results can be only applicable to previously hospitalized COVID-19 survivors with mild-to-moderate severity. In fact, critically ill COVID-19 survivors also exhibit “de novo” post-COVID pain symptoms [27]. It is probably that severely ill patients can exhibit a higher prevalence of neuropathic pain. Second, we did not collect laboratory measures, such as inflammatory biomarkers, which could be also associated with neuropathic pain. There is preliminary evidence supporting that both musculoskeletal [28] and neuropathic pain in COVID-19 survivors are associated with serological biomarkers [29]. Third, the presence of preexisting symptoms before the SARS-CoV-2 infection could be a risk factor for developing neuropathic pain. Fourth, although our results suggest that post-COVID pain involves neuropathic symptomatology in almost 25% of the individuals, post-COVID pain has been also classified as nociplastic pain [30]. It is likely that post-COVID pain consists of a complex disorder involving different mechanisms at the same time.

## 5. Conclusions

The prevalence of neuropathic pain symptoms in previously hospitalized COVID-19 survivors exhibiting “de novo” post-COVID pain is almost 25%. Furthermore, the presence of neuropathic pain was associated with more anxiety and kinesiophobia levels, but only kinesiophobia level was significantly associated, explaining 12.8% of the variance of the S-LANSS score.

## Figures and Tables

**Table 1:** Baseline outcomes (mean ± SD) of the sample.

Variable	Total sample (*n* = 77)	S-LANSS ≥ 12 points (*n* = 19)	S-LANSS < 12 points (*n* = 58)
Demographic variables			
Age (years)	60.0 ± 11.5	60.0 ± 8.0	60.2 ± 12.5
Height (m)	1.69 ± 0.09	1.68 ± 0.09	1.69 ± 0.09
Weight (kg)	75.8 ± 15.4	72.6 ± 12.7	74.8 ± 16.1
Pain and sensitization-related variables			
Time with symptoms (months)	6.0 ± 0.8	5.6 ± 0.7	6.0 ± 0.9
Pain intensity (0–10)	5.4 ± 1.8	5.8 ± 1.8	5.3 ± 1.7
S-LANSS (0–24)^*∗*^	7.7 ± 6.5	15.6 ± 2.8	4.2 ± 4.1
Psychological variables			
HADS-A (0–21)	5.8 ± 4.4	7.2 ± 4.1	5.3 ± 4.4
HADS-D (0–21)	5.7 ± 4.7	6.5 ± 4.4	5.4 ± 4.8
Cognitive and health-related variables			
PSC (0–52)^*∗*^	16.1 ± 13.1	23.2 ± 11.5	14.0 ± 12.9
TSK-11 (0–44)^*∗*^	24.2 ± 9.1	27.5 ± 10.1	23.2 ± 8.5

HADS, hospital anxiety and depression scale; PSQI, Pittsburgh Sleep Quality Index; PCS, Pain Catastrophizing Scale; TSK-11: Tampa Scale for Kinesiophobia. ^*∗*^Significant differences between individuals according to the CSI score (Student's *t*-test, *P* < 0.01).

**Table 2:** Pearson-product moment correlation matrix between sociodemographic, psychological, neurophysiological, and clinical characteristics.

	1	2	3	4	5	6	7	8	9
1. Age									
2. Weight	n.s.								
3. Height	n.s.	0.521^*∗∗*^							
4. Time with symptoms	n.s.	n.s.	n.s.						
5. Mean pain intensity	n.s.	n.s.	n.s.	n.s.					
6. HADS-A	n.s.	n.s.	n.s.	n.s.	n.s.				
7. HADS-D	n.s.	n.s.	n.s.	n.s.	0.301^*∗*^	0.867^*∗∗*^			
8. PCS	n.s.	n.s.	n.s.	n.s.	0.321^*∗*^	0. 672^*∗∗*^	0.619^*∗∗*^		
9. TKS-11	n.s.	n.s.	n.s.	n.s.	0.429^*∗∗*^	0.409^*∗∗*^	0.365^*∗∗*^	0.639^*∗∗*^	
10. S-LANSS	n.s.	n.s.	n.s.	0.262^*∗*^	n.s.	0.275^*∗*^	n.s.	n.s.	0.291^*∗*^

S-LANSS, Self-report Leeds Assessment of Neuropathic Symptoms and Signs; HADS, Hospital Anxiety and Depression Scale; PCS, Pain Catastrophizing Scale; TSK-11: Tampa Scale for Kinesiophobia. ^*∗*^*P* < 0.05; ^*∗∗*^*P* < 0.01.

## Data Availability

The raw data used to support the findings of this study are available from the corresponding author upon request.

## Supplementary Materials

Supplementary Materials The supplementary file shows the Strengthening the Reporting of Observational Studies in Epidemiology (STROBE) guideline. *Supplementary Materilas*
